# Sex and season influence behaviour and physiology of lake trout following angling

**DOI:** 10.1093/conphys/coae041

**Published:** 2024-07-05

**Authors:** Bradley E Howell, Giulio Navarroli, Simon W DePasquale, Steven J Cooke, Caleb T Hasler

**Affiliations:** Fish Biology and Conservation Laboratory, Department of Biology, The University of Winnipeg, 515 Portage Avenue, Winnipeg, MB R3B 2E9, Canada; Fish Biology and Conservation Laboratory, Department of Biology, The University of Winnipeg, 515 Portage Avenue, Winnipeg, MB R3B 2E9, Canada; Fish Biology and Conservation Laboratory, Department of Biology, The University of Winnipeg, 515 Portage Avenue, Winnipeg, MB R3B 2E9, Canada; Fish Ecology and Conservation Physiology Laboratory, Department of Biology and Institute of Environmental and Interdisciplinary Science, Carleton University, 1125 Colonel By Drive, Ottawa, ON K1S 5B6, Canada; Fish Biology and Conservation Laboratory, Department of Biology, The University of Winnipeg, 515 Portage Avenue, Winnipeg, MB R3B 2E9, Canada

**Keywords:** Barotrauma, catch-and-release, mortality, reflex, stress

## Abstract

Catch-and-release angling exposes fish to challenges that may result in sub-lethal effects or mortality. Lake trout (*Salvelinus namaycush*) undergo high rates of release because of size-based harvest regulations or voluntary angler behaviour. Here, we examine short-term impairment in lake trout angled during the summer (*n* = 74) and fall spawning period (*n* = 33) to inform best practices for angling. Immediately following capture or 0.5 h post-capture, fish underwent reflex and barotrauma assessments, and a small blood sample was collected. Fish were also fitted with an externally mounted biologger equipped with depth, temperature and tri-axial acceleration sensors, that was tethered to allow retrieval of the logger after 14 min. In the summer, reflex impairment and barotrauma at 0 and 0.5 h were significantly correlated. Loss of orientation and bloating were the most observed indicators. Larger fish and those captured at increased depth had higher barotrauma scores, while prolonged fight times decreased the barotrauma score regardless of sampling time. Plasma cortisol, lactate and glucose increased 0.5 h after capture, and extracellular and intracellular pH decreased, all signs that angling was inducing a metabolic response. However, no relationships were found between blood indices and mortality (18.9%). The time required to reach maximum depth after release was longer for fish with increased air exposure but shorter for those with longer fight times. During the fall, fish displayed no mortality or reflex impairment. Anal prolapse was the most observed indicator of barotrauma but only observed in females. Blood indices were most altered 0.5 h after capture, with increased cortisol values for fish that were female, particularly large or captured at deeper depth. Locomotor activity was highest for males and increased with depth. Together, our findings suggest that the effects of catch-and-release angling may be dependent on several factors, including sex, season and angling depth.

## Teaser

We examined impacts of catch-and-release angling on lake trout angled during the summer and fall spawning season. We measured reflexes, blood indices, and, briefly, post-release locomotor activity. Temperature, season, angling depth, and sex influenced many responses. Anglers should employ context-specific practices to prevent negative outcomes when angling for lake trout.

## Introduction

In recreational fisheries, angled fish are often released (i.e. catch-and-release; C&R) to either comply with regulations or as a result of voluntary angler behaviour (Arlinghaus *et al.,* 2007). The concept behind C&R is that by releasing fish, they will have the potential to grow, reproduce and be recaptured. However, this tenet assumes that changes in injuries, behaviour and physiology are minimal and that fish survive the C&R event (Cooke and Schramm, 2007). Acute behavioural and physiological consequences are possible for fishes that are captured by angling, and these effects can compound with abiotic factors to affect survival (Gingerich *et al.,* 2007; McLean *et al.,* 2020; Van Leeuwen *et al.,* 2021). Numerous studies have attempted to quantify angling-related mortality (Muoneke and Childress, 1994; Arlinghaus *et al.,* 2007). Factors such as hooking location, increased angling depth, warm water temperatures and extended fight and handling times have been identified as significant mortality predictors (Bartholomew and Bohnsack, 2005; Arlinghaus *et al.,* 2007). Quantifying the many factors that influence fish condition and survival following C&R is imperative for fisheries conservation (Brownscombe *et al.,* 2014a).

Coupling visual observations with physiological biomarkers allows for a comprehensive assessment of the extent of sub-lethal impairments as well as the likelihood that a fish will survive an angling event (Arlinghaus *et al.,* 2009; Twardek *et al.,* 2018). For example, reflex impairment scoring is an inexpensive, field-based assessment that measures fish vitality by examining innate reflexes (Davis, 2007; Davis, 2010). The total amount of reflexes that are present (or absent) can be an indicator of the fish’s physiological state and potentially predictive of the likelihood of survival (Davis, 2010; Raby *et al.,* 2012). Additionally, some fish can experience pressure-related injuries known as barotrauma (Carlson, 2012). Fish are either physostomous and have a pneumatic duct that allows expulsion of excess gas through the oesophagus, or physoclistous and have an oval chamber and diffuse excess gas through a capillary mesh that supplies blood to the swim bladder (Saunders, 1953; Ferguson, 1989; St John, 2003). Physoclistous species, compared to physostomous species, are thought to be more susceptible to rapid changes in external pressure due to their slower process of gas release. Fish that are undergoing barotrauma display various external symptoms (Rummer and Bennett, 2005), and scoring of these can be done to determine severity (Carlson, 2012). Furthermore, physiological biomarkers such as hormones, metabolites or ions can be assayed using small non-lethal blood samples and are often measured in fish following angling (Sopinka *et al.,* 2016). Physiological samples taken from wild fish following angling provide insight into the general stress response and metabolic demands, and allow researchers to understand possible correlates of post-release survival, such as air exposure and fight duration (Skomal, 2007). Overall, visual observation and measurement of physiological biomarkers following angling are necessary for assessing the degree to which fish are affected by angling.

Using electronic tags to assess C&R consequences has also become a prevalent tool given their potential to increase realism of experimental studies (Donaldson *et al.,* 2008). For example, understanding the energetic cost of angling is important in determining fish impairment and recovery (Watson *et al.,* 2020). Biologgers equipped with acceleration sensors have been used to answer a wide breadth of questions relating to fine-scale movements, kinematics and metabolic rate in both fresh and saltwater fishes (Gleiss *et al.,* 2010; Brown *et al.,* 2013; Brownscombe *et al.,* 2014b; Metcalfe et al., 2015; Bouyoucos *et al.,* 2017). By using acceleration-sensing tags, overall dynamic body acceleration (ODBA, an integrated measure of 3D body motion) can be estimated and used as a proxy for energy expenditure (Halsey *et al.,* 2009). ODBA is the sum of absolute values of dynamic accelerations from three separate spatial axes (Wilson *et al.,* 2006). Research exploring C&R with biologgers equipped with acceleration sensors has validated ODBA as a useful tool for understanding how fish behaviour can be impaired after a fish has been released (Lennox *et al.,* 2018; Griffin *et al.,* 2022). Studies using ODBA to measure locomotion in both summer (Chhor *et al.,* 2022a; Madden *et al.,* 2024) and winter (LaRochelle *et al.,* 2021; Bieber *et al.,* 2022) illustrate that fishes may be affected by an angling event with the level of effect mediated by environmental conditions.

Lake trout (*Salvelinus namaycush;* a physostomous species) are a sportfish targeted by anglers and spend much of their life in cool, deep water. Seasonality can have a major impact on the degree to which fish are affected by angling. The majority of C&R occurs during the open-water season (i.e. spring, summer and fall in temperate climates), which is characterized by high air and water temperatures (Cowie and Ridgway, 2023; Sass *et al.,* 2023). The open-water season is also a time when fish are more active and metabolic processes are faster (Johnston and Dunn, 1987; Anderson *et al.,* 1998). Environmental conditions can amplify physiological disturbances from C&R and increase susceptibility to reflex impairment, barotrauma impairment or mortality (Wilkie *et al.,* 1996; Cooke and Suski, 2005; Gingerich *et al.,* 2007; Schreer *et al.,* 2009; Donaldson *et al.,* 2014; Logan *et al.,* 2019). For fall spawning fish such as lake trout, behavioural changes occur during the spawning period, as mature fish move towards shallower waters (Crisp and Carling, 1989; Esteve *et al.,* 2008; Binder *et al.,* 2015; Johnson *et al.,* 2018). Angling during the spawning period is a concern because behavioural and physiological impairment might prevent fish from engaging in typical spawning behaviours and thus decrease individual fecundity (Van Overzee and Rijnsdorp, 2015). Using genetic analysis, Richard *et al.* (2013) found that large Atlantic salmon (*Salmo salar*) produced less offspring when angled. Therefore, understanding how seasonality influences the effects of C&R on lake trout is important for developing evidence-based angler education materials and fishing regulations.

In our study, we aimed to determine whether C&R angling induced short-term changes in reflexes, physiology and behaviour of lake trout angled during the summer and fall spawning period. We assessed reflexes, barotrauma and blood parameters at 0 or 0.5 h post-capture (i.e. baseline vs. post-capture response). After sampling, a biologger (equipped with depth, temperature and tri-axial acceleration sensors) was quickly attached, which allowed us to monitor post-release activity for 14 min. Additionally, because our angling practices varied, we also examined interactions of variables that may contribute to impairment and mortality (e.g. fight and air exposure duration). Lake trout were expected to display a more pronounced response to angling during the summer (i.e. higher reflex impairment and greater levels of physiological alteration) because of elevated water temperatures and presence at deeper depths. We also expected that because fall fish would be reproductively mature, a different response to angling may occur. Post-release activity was expected to be higher for fish displaying barotrauma because of prolonged attempts to return to depth. Our study may contribute to refining best practices for angling lake trout during warm conditions and during the spawning period.

## Materials and Methods

### Study location

We angled lake trout from June 23 to July 20 and October 1 to 6, 2022, on Clearwater Lake, Manitoba, Canada (54.0570° N, 101.0564° W). The lake has a surface area of 593 km^2^ and an average depth of 13.1 m (maximum depth = 39 m). The clarity of the lake is 10 m. During the summer angling period, air temperatures ranged from 13 to 30°C (mean = 22.05°C) (Environment and Climate Change Canada, 2022) and surface water temperatures were between 15.9 and 21.3°C (mean = 18.6°C) (Pro20 model; YSI Inc., Yellow Springs, OH, USA; range = −5 to 55°C, accuracy = ±0.3°C). Surface dissolved oxygen levels were between 4.2 and 8 mg/L (mean = 6.4 mg/L), and we angled fish between 10 and 42 m (mean = 26 m). At 30 m below the surface, water temperatures were between 5.7 and 10.4°C (mean = 7.8°C), and dissolved oxygen levels were between 5 and 11.4 mg/L (mean = 8.9 mg/L) (see Supplementary Fig. S1 for full vertical profile). During the fall angling, air temperatures ranged from 0 to 22°C (mean = 12.9°C) and surface water temperatures were between 10.8 and 14.4°C (mean = 13.2°C). Surface dissolved oxygen levels were between 11.2 and 13.4 mg/L (mean = 12.1 mg/L), and we angled fish between 1.5 and 13.7 m (mean = 3.4 m). At 6 m below the surface, water temperature was 13.4°C, and dissolved oxygen was 2.9 mg/L.

### Fish capture and holding

Our research was conducted in accordance with an animal care protocol approved by the Animal Care Committee of The University of Winnipeg (Animal Use Protocol #AE10491), following guidance by the Canadian Council on Animal Care. We also had a Provincial Scientific Collection (General) Permit (#22758865). Angling gear consisted of spinning rods spooled with 13.61-kg braided line and a 1.22-m 5.44-kg fluorocarbon leader on a size 35 reel. We rigged rods with either spoons, tubes, vertical jigs, crankbaits, jerkbaits or meat rigs with a single size 1/0 barbless treble hook depending on angler preference and water depth. The angling location on the lake varied, and we recorded water depth, temperature and dissolved oxygen at each sample site from the boat.

Six experienced anglers captured the fish. Once a fish strike was felt, the angler quickly raised their fishing rod to set the hook and reeled in the fish. We recorded with a stopwatch the time between hooking the fish and the fish clearing the water (i.e. being landed in a net), and the amount of time the fish was exposed to air prior to being placed into a 378-l stock tank filled with fresh surface lake water. Upon capture, we immediately unhooked the fish and noted the hooking location and level of bleeding. Bleeding was scored on a three-point scale (adapted from Falk *et al.,* 1974): 0 score, none, no external bleeding near the hook entry point; 1 score, slight, a small amount of bleeding localized near the hook entry point; and 2 score, flowing, blood surrounding and obscuring the hook entry point. We also measured fish for total length (±1 mm) and mass (±0.01 kg) before placing them into the stock tank. Water temperature and dissolved oxygen within the tub fluctuated and was changed between fish. One group of fish immediately (0 h) underwent behavioural assessments, blood sampling (see below) and then post-release accelerometry (to provide ‘baseline’ values indicative of a quick fish release), while another group was held in the stock tank for 0.5 h prior to sampling (to provide post-capture values when blood indices should be peaking) (Milligan and Wood, 1986).

### Behavioural assessments

Although fish were in the tank, reflex and barotrauma assessments were completed. We completed reflex assessments following previously established methods (e.g. Howell *et al.,* 2023). Our assessment included the following metrics: (1) tail grab, burst swimming response to caudal peduncle grab; (2) body flex, attempted escape when held out of the water by midsection; (3) head complex, opening of jaws in normal ventilation pattern when held out of water; (4) vestibular–ocular response, tracking of eye to remain level when rotated horizontally and held out of water; (5) orientation, vertical alignment after being placed upside down in a holding bin. We calculated the overall reflex impairment score for each individual as the counted total of the five reflexes that were impaired, with scores for each reflex recorded as either 0 (unimpaired) or 1 (impaired) (as per Davis, 2010).

To determine the extent at which barotrauma occurred in angled fish, we completed a barotrauma assessment (see Althoff *et al.,* 2021; Howell *et al.,* 2023) in the same tank filled with fresh lake water. We checked for the presence of the following: (1) oral organ eversion, gastric herniation into the buccal cavity; (2) exophthalmia, bulging eyes; (3) bloating, overinflation of the midsection; (4) anal organ eversion, prolapsed anus; and (5) haemorrhaging, redness in the mouth/gills/fins/anus. We calculated the overall barotrauma score for each individual as the counted total of the five reflexes that were impaired. A single observer (B.E.H.) completed both assessments for all fish to limit variation between samplers. Both reflex and barotrauma assessments were completed in under 120 s.

### Blood sampling

Following reflex and barotrauma assessments, we held fish ventral-side up with gills submerged in the water within the tank. Blood (1.5 ml) was then drawn via caudle puncture with a 10-ml lithium heparin vacutainer and 21-G needle. Less than 10% of total blood volume was removed (Lawrence *et al.,* 2020). We then immediately centrifuged blood at 6000*g* for 3 min to separate plasma from other blood components. Plasma was allocated into three separate 0.6-ml vials before being placed into a vapour shipper charged with liquid nitrogen along with the remaining red blood cells. Once transported back to the laboratory, we stored plasma and other blood component samples at −80°C.

In the laboratory, we determined cortisol concentrations in blood plasma using commercially available enzyme-linked immunosorbent assay (ELISA) kits (#402710; Neogen, Lexington, KY, USA) and a microplate spectrophotometer (SpectraMax i3; Molecular Devices, San Jose, CA, USA). The ELISA kits have been previously used for analysis of salmonid plasma samples (Raby *et al.,* 2015; Howell *et al.,* 2023). We ran samples in triplicate at a dilution factor of 200 after having completed a dilution series of 25, 50, 100, 200 and 400 to choose the appropriate factor based on a standard curve. Intra-assay variations (%CV) for summer and fall were 4.37% and 7.27%, respectively; inter-assay variations were 9.92% and 23.23%. We determined plasma lactate and glucose concentrations following the enzymatic methods of Lowry and Passonneau (1972) with triplicates and a dilution factor of 3.75 for lactate. Intra-assay variations for lactate were 5.22% and 5.48%; inter-assay variations were 6.02%. Intra-assay variations for glucose were 9.43% and 6.69%; inter-assay variation was 13.94%. We measured extracellular pH from thawed plasma and intracellular pH from lysed red blood cells that went through five freeze–thaw cycles (Mullen *et al.,* 2020; Howell *et al.,* 2023) (HI98165 pH Meter; HANNA Instruments, Woonsocket, RI, USA).

### Post-release behaviour

We released surviving fish with a coloured T-bar anchor tag inserted on the left side of the dorsal fin after they had been sampled to ensure each individual was only sampled once during the study. The vigour of release was scored on a three-point scale: 0 or poor, lethargic movement and lack of consistent tail beats; 1 or good, regular movement and consistent tail beats; and 2 or excellent, energetic movement and fast-paced tail beats. No fish were recaptured.

To evaluate post-release locomotion, we attached biologgers equipped with depth, temperature and tri-axial acceleration sensors (Axy 5 S Depth; TechnoSmArt, Rome, Italy; sampling rate: acceleration = 10 Hz, temperature/depth = 1 Hz; resolution: acceleration = 8-bit, temperature = 0.1°C, depth = 5 cm, G scale = 8; size: 36 × 14 × 9 mm, 6.03 g) around the midsection of the fish using a harness that we made by bonding the biologger to a waterproof 3D-printed plate (PET-G, Sakata 3D filaments, Granada, Spain; Size: 36 × 22 × 2 mm, 0.96 g) with marine epoxy and then threading this onto a section of Velcro tape (One-Wrap Roll; Velcro Brand, Manchester, NH, USA; length = 35–68 cm, width = 2 cm). We used varying lengths of Velcro tape to accommodate differing fish girths. We attached the harness while fish were held out of water with Velcro tape wrapped once around the fish anterior to the dorsal fin, positioning the accelerometer on the lateral side above the left pectoral fin (see Supplementary Fig. S2). Harness attachment took no longer than 30 s, and Velcro straps were attached as to not inhibit the normal range of motion to reduce the impact of the harness on fish behaviour. The accelerometer harness was connected to a rod and reel spooled with 13.61-kg braided line using a snap swivel clipped through a reinforced hole in the Velcro tape (see Chhor *et al.,* 2022a; Chhor *et al.,* 2022b). Fish were released by hand at the side of the boat and allowed to swim without resistance for 15 min on a “free-spool” before we retrieved the accelerometer harness by tugging the line, which released the Velcro. To ensure the tag had not fallen off during the 15-min testing period, we checked for the presence of the fish on the end of the line before retrieving the accelerometer by feeling for motion and weight. We observed no entanglement of the line.

Prior to data analysis, we trimmed raw acceleration data to include only the first 14 min to standardize the length of recorded activity between all individuals. We calculated static acceleration by passing a 2-s smoother over each axis with the *rollmean* function in the *zoo* package (Zeileis and Grothendieck, 2005) and converted the values to degrees by multiplying values by 180*π*^−1^ (Brownscombe *et al.,* 2013). We then calculated dynamic acceleration (*g*) by subtracting static acceleration from raw acceleration values for each axis. Next, we calculated ODBA by summing the absolute values of dynamic acceleration in each axis (Lennox *et al.,* 2018).

### Data analysis and statistics

We completed all statistical analyses in R version 4.1.2 (R Core Team, 2021) with level of significance assessed at *α* ≤ 0.05 unless otherwise stated, and *P* values rounded to the nearest thousandth. We performed separate Wilcoxon tests to test whether blood indices differed across recovery times in the summer, for cortisol, lactate, glucose, extracellular pH and intracellular pH using the *rstatix* package due to the data not meeting assumptions of homogeneity and normality (Kassambara, 2021). Homogeneity of variance was assessed using Levene’s test (*cars* package; Fox and Weisberg, 2019), and normality was assessed using the Shapiro–Wilk test (*stats package*; R Core Team, 2021). Outliers were identified using Grubbs’ test (*outliers* package; Komsta, 2022), and effect size was calculated using Wilcoxon effect size (*rstatix* package; Kassambara, 2021). A Bonferroni correction was used to account for multiple statistical tests being performed, and significance was therefore determined at *α* = 0.01. To test whether blood indices differed across recovery times or sexes in the fall, separate two-way ANOVAs were performed (*stats* package; R Core Team, 2021). When the model assumption tests failed for cortisol and lactate, we used a log transformation prior to the two-way ANOVA. If an interaction was not significant, it was removed, and the ANOVA was re-run.

We used binary logistic regression models to determine the relationship between cortisol, lactate, glucose, extracellular pH and intracellular pH at both recovery times and the occurrence of mortality in the summer (*stats* package; R Core Team, 2021). Mortality was regressed against each blood metric independently for both recovery times. A Bonferroni correction was used to account for multiple statistical tests, and significance was determined at *α* = 0.01. Linear models were used to determine the effect of length, fight time, air exposure, depth, reflex score and barotrauma score on cortisol, lactate, glucose, extracellular pH and intracellular pH 0.5 h post-angling in the summer and fall (*stats* package; R Core Team, 2021). Blood indices were used as the response variable in each model. Due to multiple tests being conducted on the same samples, a Bonferroni correction was used to account for multiple statistical tests and significance was determined at *α* = 0.008 (summer) and *α* = 0.01 (fall).

We used binary multiple regression models to determine the effects of recovery time, length, fight time, air exposure and depth on whether any reflex or barotrauma impairment was observed (*stats* package; R Core Team, 2021). A Bonferroni correction was used to account for multiple statistical tests, and significance was determined at *α* = 0.008 (summer) and *α* = 0.007 (fall). Interaction between reflex and barotrauma scores in the summer was tested with Kendall correlation (*stats* package; R Core Team, 2021). Interaction between reflex and barotrauma scores in the fall was not analysed due to no reflex impairment and minimal barotrauma signs.

Generalized additive mixed models (GAMMs) were used to determine interactions between ODBA and angling depth in the summer, and angling depth and sex in the fall (*gamm4* package; Wood and Scheipl, 2020). The interaction between ODBA and angling depth in the fall was not analysed due to most fish being caught at similar depth (~2.3 m). Time post-capture, recovery time, fish length, fight time and air exposure time were initially included in the model but removed due to multi-collinearity. Sex was not included in the summer GAMM because it was not possible to determine sex during that time of year. Linear models were used to determine the effects of fight time and air exposure on the time it took lake trout to reach their maximum depth following release (*stats* package; R Core Team, 2021).

## Results

### Summer angling

We caught 74 lake trout during the summer, and these fish had a mean (±SD) total length of 611 ± 113 mm with a range of 513–1073 mm. The mean weight of these fish was 2151 ± 1788 g with a range of 1090–10 120 g. The mortality rate was 18.9%. Of the 14 mortalities, one lake trout died immediately (i.e. within seconds of landing), eight were moribund during the 0-h sampling and five died within 0.5 h of holding.

**Figure 1:**
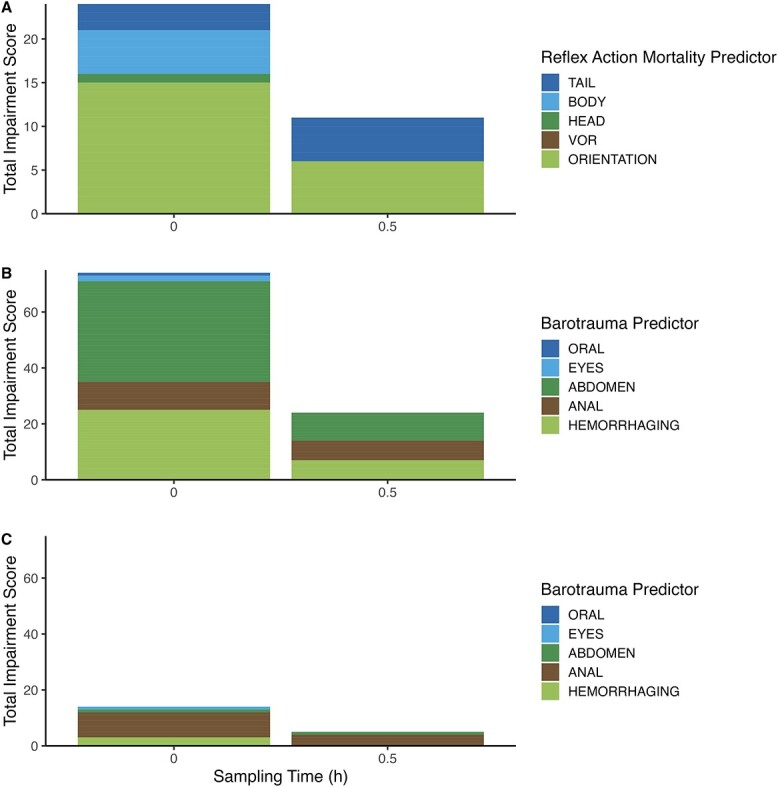
Totalled impairment scores for (A) summer reflex, (B) summer barotrauma and (C) fall barotrauma predictors in lake trout (*S. namaycush*) sampled following angling. Reflex metrics include (1) tail grab, (2) body flex, (3) head complex, (4) vestibular–ocular response, and (5) orientation. Barotrauma metrics include (1) oral organ eversion, (2) exophthalmia, (3) bloating, (4) anal organ eversion, and (5) haemorrhaging.

Lake trout displayed signs of reflex and barotrauma-related impairment, with highest impairment scores for both assessments immediately following capture (Fig. 1). Loss of orientation was the most observed reflex impairment across sampling times (0 h = 31.9% of fish, 0.5 h = 22.2% of fish). Bloating of the abdomen was the most observed barotrauma impairment across sampling times (0 h = 76.5% of fish, 0.5 h = 37% of fish) (mean angling depth = 26 m). Of the 74 fish caught, 4% were caught between 10 and 20 m, 50% were caught between 20 and 30 m, and 46% were caught deeper than 30 m. One fish displayed oral organ eversion immediately after capture and died within 5 min. Sampling time, total length, fight time, air exposure and depth did not influence reflex scores, but fish total length, fight time and angling depth each influenced barotrauma scores (Table 1). Reflex impairment and extent of barotrauma were also positively correlated (Kendall rank correlation: *z* = 3.77, *r* = 0.44, *P* < 0.05).

**Table 1:** Summary of binary multiple logistic regression models assessing the effects of sampling time, fish total length, fight time, air exposure time and water depth on whether lake trout (*S. namaycush*) showed impairment for any of five reflex or barotrauma metrics assessed post-angling in the summer and fall

Season	Assessment	Variable	Estimate	SE	*z* value	*df*	*P*
Summer	Reflex	Sampling time, 0 h	−0.23	0.83	−0.28	71	0.78
		Sampling time, 0.5 h	0.09	0.86	0.10	71	0.92
		Fish total length	0.01	0.01	1.55	71	0.12
		Fight time	0.01	0.01	0.74	71	0.46
		Air exposure	0.00	0.01	0.72	71	0.47
		Angling depth	−0.00	0.05	−0.04	71	0.97
Summer	Barotrauma	Sampling time, 0 h	2.85	1.21	2.35	71	0.02
		Sampling time, 0.5 h	0.50	1.04	0.48	71	0.63
		**Fish length**	**0.04**	**0.01**	**3.04**	**71**	**0.00**
		**Fight time**	**−0.06**	**0.02**	**−3.05**	**71**	**0.00**
		Air exposure	0.01	0.01	0.71	71	0.48
		**Angling depth**	**0.12**	**0.07**	**2.66**	**71**	**0.01**
Fall	Barotrauma	Sampling time, 0 h	0.13	1.51	0.08	29	0.93
		Sampling time, 0.5 h	−0.82	1.72	−0.48	29	0.63
		Fish total length	−0.00	0.02	−0.04	29	0.97
		Fight time	−0.05	0.08	−0.70	29	0.49
		Air exposure	−0.04	0.04	−1.23	29	0.22
		Angling depth	0.28	0.32	0.87	29	0.39
		Sex	2.11	1.84	1.15	29	0.25

Statistical significance was determined at Bonferroni-corrected *α* = 0.008 for summer and *α* = 0.007 for fall. Significance is bolded.

**Figure 2:**
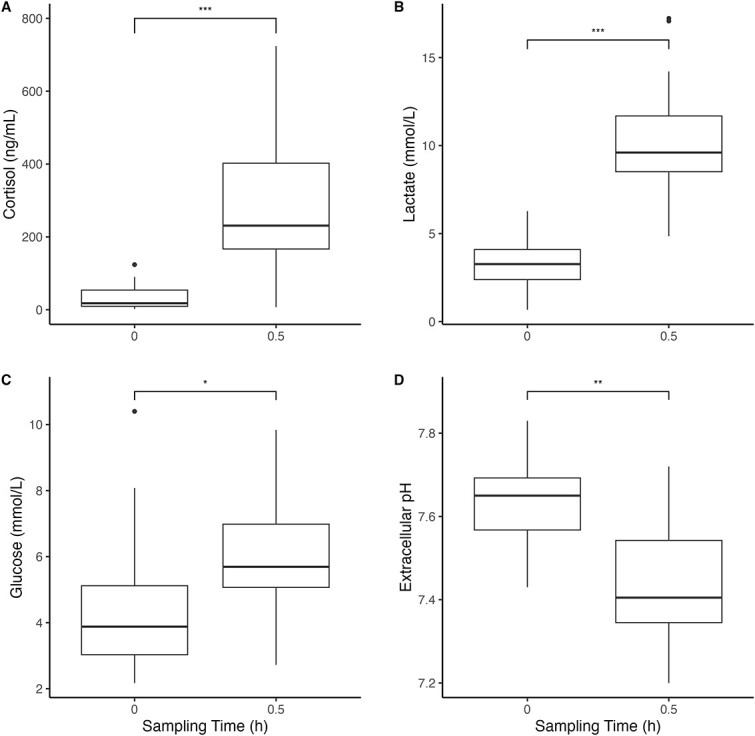
Concentrations of (A) plasma cortisol, (B) plasma lactate, (C) plasma glucose and (D) extracellular pH in lake trout (*S. namaycush*) sampled at 0 (*n* = 32) or 0.5 h (*n* = 26) following summer angling. Thick black horizontal lines denote median values, boxes contain all data within the 25th and 75th quartiles, whiskers show the range of data and outliers are depicted as black dots. Asterisks above horizontal brackets denote effect size (^*^^*^^*^Cohen’s *d* ≥ 0.8, ^*^^*^Cohen’s *d* ≥ 0.5, ^*^Cohen’s *d* ≥ 0.2).

Angling induced changes in physiological stress indices. Comparing the 0.5-h post-capture data to the 0-h baseline data, cortisol increased by 89.8% (Wilcox: *z* = −6.08, *P* < 0.001, Cohen’s *d* = 0.80), lactate increased by 67.5% (Wilcox: *z* = −6.41, *P* < 0.001, Cohen’s *d* = 0.84), glucose increased by 27.7% (Wilcox: *z* = −3.35, *P* < 0.001, Cohen’s *d* = 0.44), extracellular pH decreased by 2.6% (Wilcox: *z* = 4.76, *P* < 0.001, Cohen’s *d* = 0.62) and intracellular pH decreased by 1.1% (Wilcox: *z* = 4.23, *P* < 0.001, Cohen’s *d* = −0.56) (Fig. 2). No relationships were found between the blood indices at either sampling time and likelihood of mortality (Table 2). None of the variables (i.e. total length, fight time, air exposure, angling depth, reflex or barotrauma score) explained variation in any of the blood indices (Table 3).

**Table 2:** Summary of binary logistic regression models assessing the effects of cortisol (ng/mL), lactate (mmol/L), glucose (mmol/L), extracellular pH or intracellular pH of lake trout (*S. namaycush*) on whether a fish survived the angling event or not (fish sampled at 0 or 0.5 h post-angling in the summer)

Time	Blood metric	Estimate	SE	*z* value	*df*	*P*
0	Cortisol	0.05	0.03	1.46	31	0.14
	Lactate	−3.31	1.70	−1.95	31	0.05
	Glucose	−0.95	0.51	−1.87	31	0.06
	Extracellular pH	−13.97	8.30	−1.68	31	0.09
	Intracellular pH	−30.98	21.62	−1.43	31	0.15
0.5	Cortisol	−0.00	0.00	−1.14	25	0.26
	Lactate	−0.13	0.26	−0.49	25	0.62
	Glucose	0.09	0.40	0.23	25	0.82
	Extracellular pH	−19.80	12.21	−1.62	25	0.11
	Intracellular pH	23.03	16.96	1.36	25	0.17

Statistical significance was determined at a Bonferroni-corrected *α* = 0.01 and is bolded.

**Table 3:** Summary of model selection results based on linear models predicting the cortisol, lactate, glucose, extracellular pH and intracellular pH concentrations of lake trout (*S. namaycush*) sampled at 0.5 h post-angling in the summer

Blood metric	Variable	Estimate	SE	*df*	*t*	*P*
Cortisol	(Intercept)	−610.32	771.67	19	−0.79	0.44
	Fish length	1.08	1.31	19	0.82	0.42
	Fight time	−4.08	3.48	19	−1.17	0.26
	Air exposure	−0.80	0.94	19	−0.85	0.41
	Angling depth	22.80	8.91	19	2.56	0.02
	Reflex	−131.95	74.57	19	−1.77	0.09
	Barotrauma	10.78	55.78	19	0.19	0.85
Lactate	(Intercept)	22.95	11.46	19	2.00	0.06
	Fish length	−0.03	0.02	19	−1.75	0.10
	Fight time	0.03	0.05	19	0.67	0.51
	Air exposure	0.00	0.01	19	0.33	0.74
	Angling depth	0.15	0.13	19	1.12	0.28
	Reflex	0.92	1.11	19	0.83	0.42
	Barotrauma	0.84	0.83	19	1.01	0.33
Glucose	(Intercept)	21.19	7.10	19	2.99	0.01
	Fish length	−0.03	0.01	19	−2.15	0.05
	Fight time	0.07	0.03	19	2.11	0.05
	Air exposure	−0.01	0.01	19	−1.02	0.32
	Angling depth	−0.09	0.08	19	−1.09	0.29
	Reflex	−0.02	0.69	19	−0.03	0.98
	Barotrauma	0.23	0.51	19	0.44	0.66
Extracellular pH	**(Intercept)**	**6.78**	**0.49**	**19**	**13.89**	**0.00**
	Fish length	0.00	0.00	19	1.36	0.19
	Fight time	−0.00	0.00	19	−0.81	0.43
	Air exposure	0.00	0.00	19	0.66	0.52
	Angling depth	0.00	0.01	19	0.76	0.46
	Reflex	−0.08	0.05	19	−1.74	0.10
	Barotrauma	−0.04	0.04	19	−1.24	0.23
Intracellular pH	**(Intercept)**	**6.86**	**0.20**	**19**	**34.35**	**0.00**
	Fish length	0.00	0.00	19	1.18	0.25
	Fight time	−0.00	0.00	19	−0.20	0.84
	Air exposure	−0.00	0.00	19	−0.30	0.77
	Angling depth	−0.00	0.00	19	−0.03	0.98
	Reflex	−0.02	0.02	19	−0.81	0.43
	Barotrauma	−0.03	0.01	19	−2.22	0.04

Statistical significance was determined at Bonferroni-corrected *α* = 0.008 and is bolded.

**Figure 3:**
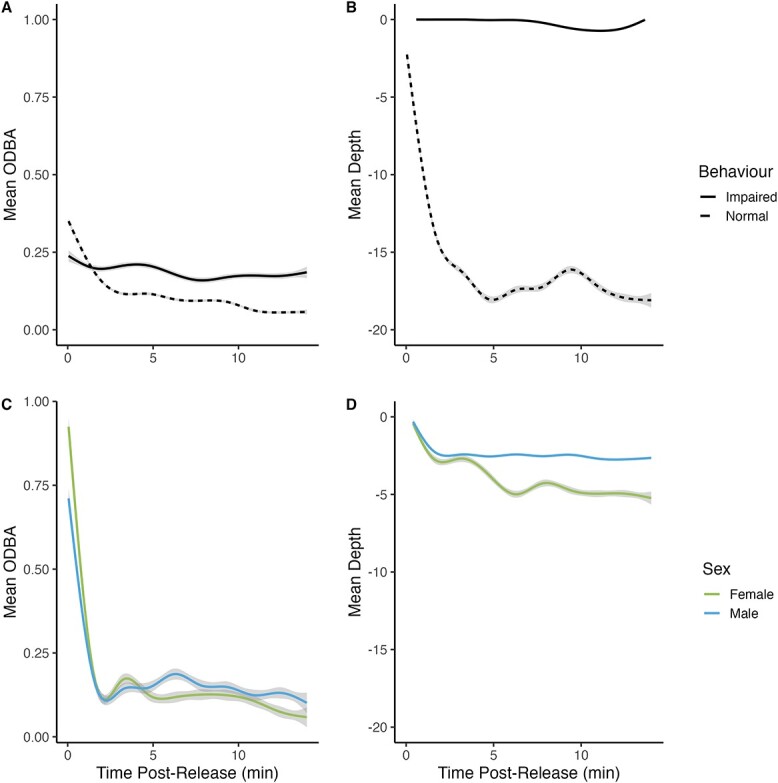
Mean (A) ODBA and (B) depth use over time post-capture by lake trout (*S. namaycush*) exhibiting normal (*n* = 9) and impaired behaviour (*n* = 42) possibly due to barotrauma injuries following summer angling. Also, mean (C) ODBA and (D) depth use over time post-capture by male (*n* = 15) and female (*n* = 15) lake trout following angling during the spawning period. Standard error is depicted as grey bands.

Upon release, 16 fish displayed a “poor” vigour score and 30 fish displayed a “good” vigour score after immediate sampling. For the 0.5-h sampling time, 3 fish displayed a “poor” score and 24 fish displayed a “good” score. Fish were observed to descend quickly to depth with high ODBA levels before reaching a desired depth and remaining there and exhibiting low ODBA levels. Longer air exposure increased the time it took to reach maximum depth while fight time decreased it (Table 4). While statistical comparisons could not be made due to low sample size, fish that remained on the surface (*n* = 9) exhibited higher mean ODBA (level of 0.202 opposed to 0.131) than those that were able to return to depth (*n* = 42) (Fig. 3). ODBA also increased with angling depth (Table 5).

**Table 4:** Summary of linear models assessing the effects of fight time and air exposure on the time it took lake trout (*S. namaycush*) to reach their maximum depth following release post-angling in the summer and fall

Season	Metric	Variable	Estimate	SE	*t*	*df*	*P*
Summer	Time to max depth	(Intercept)	10.47	206.3	5.08	37	<0.001
		**Fight time**	**−10.13**	**4.67**	**−2.17**	**37**	**0.04**
		**Air exposure**	**2.55**	**1.24**	**−2.05**	**37**	**0.05**
		**Fight × air**	**0.05**	**0.02**	**2.33**	**37**	**0.03**
Fall	Time to max depth	(Intercept)	581.22	591.36	0.98	23	0.34
		Fight time	−9.38	20.36	−0.46	23	0.65
		Air exposure	−2.25	6.40	−0.35	23	0.73
		Fight **×** air	0.14	0.23	0.62	23	0.54

Statistical significance is bolded.

**Table 5:** Summary of GAMMs showing an interaction between overall dynamic body acceleration (ODBA) and angling depth in the summer, and sex and angling depth in the fall for lake trout (*S. namaycush*) captured via angling

Season	Metric	Component	Term	Est	SE	*t*	*P*
Summer	ODBA	Param. coefficients	(Int.)	0.90	0.00	186.3	<0.001
		Component	Term	edf	Ref. *df*	*F*	*P*
		Smooth terms	**s(Depth)**	**7.83**	**7.83**	**2051**	**<0.001**
		Component	Term	Est	SE	*t*	*P*
Fall	ODBA	Param. coefficients	(Int.)	0.89	0.00	179	<0.001
			**SexMale**	**0.02**	**0.01**	**2.97**	**0.003**
		Component	Term	edf	Ref. *df*	*F*	*P*
		Smooth terms	**s(Depth)**	**8.43**	**8.43**	**1438**	**<0.001**

Statistical significance is bolded.

### Fall angling

We caught 33 lake trout during the fall, and these fish had a mean (±SD) total length of 557 ± 57 mm with a range of 462–683 mm. The mean weight of these fish was 1471 ± 445 g with a range of 830–2480 g. No mortality or reflex impairment was observed. The barotrauma score was highest immediately following capture. Prolapse of the anus was the most observed impairment across sampling times (0 h = 39.1% of fish, 0.5 h = 57.1% of fish). Aside from one male fish that exhibited exophthalmia immediately upon capture (mean angling depth = 3.37 m), fall barotrauma signs were restricted to females. Of the 33 fish caught, 7% were caught at water depths between 1 and 2 m, 83% were caught between 2 and 3 m and 10% were caught deeper than 3 m. Sampling time, total length, fight time, air exposure, depth and sex did not influence barotrauma scores (Table 1).

**Figure 4:**
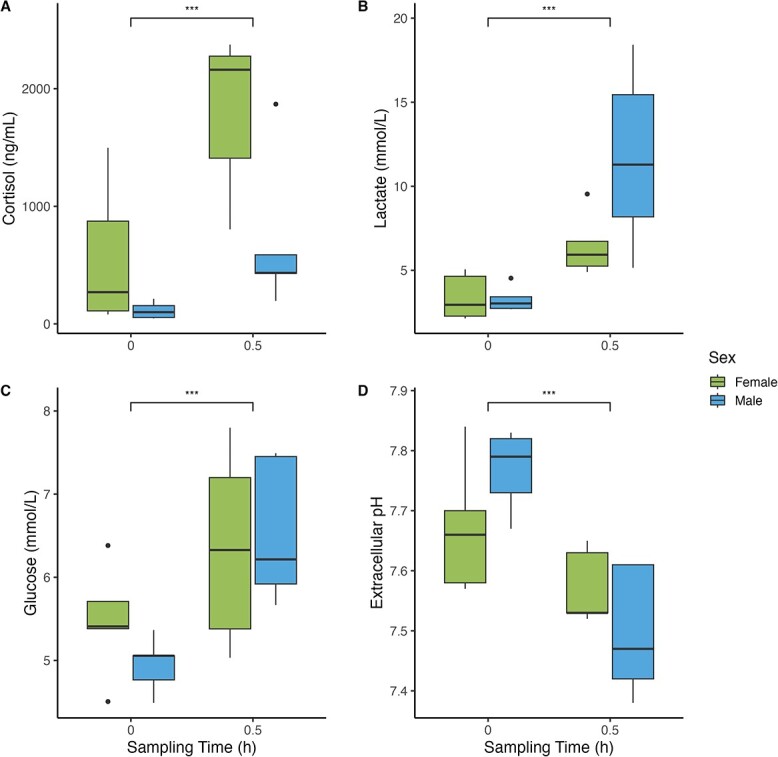
Concentrations of (A) plasma cortisol, (B) plasma lactate, (C) plasma glucose and (D) extracellular pH in lake trout (*S. namaycush*) sampled at 0 h (*n* males = 5, *n* females = 5) or 0.5 h (*n* males = 5, *n* females = 5) following fall angling. Thick black horizontal lines denote median values, boxes contain all data within the 25th and 75th quartiles, whiskers show the range of data and outliers are depicted as black dots. Asterisks above horizontal brackets denote effect size (^*^^*^^*^Cohen’s *d* ≥ 0.8, ^*^^*^Cohen’s *d* ≥ 0.5, ^*^Cohen’s *d* ≥ 0.2).

Sex and sampling time influenced blood indices (Table 6). Cortisol was 65.5% higher in females than males between sampling times (two-way ANOVA: *F*_1,17_ = 9.99, *P* < 0.01, Cohen’s *d* = 1.17) and 72.9% higher in the 0.5-h sampling time compared to the 0-h sampling time (two-way ANOVA: *F*_1,17_ = 20.55, *P* < 0.001, Cohen’s *d* = 1.68) (Fig. 4). Lactate was 63.12% higher (two-way ANOVA: *F*_1,17_ = 30.01, *P* < 0.001, Cohen’s *d* = 0.93), glucose was 19.22% higher (two-way ANOVA: *F*_1,17_ = 11.36, *P* < 0.01, Cohen’s *d* = 1.24) and extracellular pH was 2.39% lower (two-way ANOVA: *F*_1,17_ = 17.48, *P* < 0.001, Cohen’s *d* = −0.18) in the 0.5-h sampling time compared to the 0-h sampling time. Intracellular pH did not differ between sexes or sampling times. Of all the blood indices and other measured variables (aside from sex), only fish length and angling depth were found to increase cortisol (Table 7).

**Table 6:** Summary of results from two-way ANOVAs on the effects of sex and sampling time (immediately after capture or 0.5 h) on cortisol, lactate, glucose, extracellular pH and intracellular pH in lake trout (*S. namaycush*) post-angling in the fall

Blood metric	Variable	*df*	SS	MS	*F*	*P*
Cortisol	**Sex**	**1,17**	**6.86**	**6.86**	**9.99**	**<0.01**
	**Sampling time**	**1,17**	**14.12**	**14.12**	**20.55**	**<0.001**
Lactate	Sex	1,17	0.35	0.35	2.43	0.14
	**Sampling time**	**1,17**	**4.36**	**4.36**	**30.01**	**<0.01**
Glucose	Sex	1,17	0.14	0.14	0.20	0.66
	**Sampling time**	**1,17**	**7.64**	**7.64**	**11.36**	**<0.01**
Extracellular pH	Sex	1,17	0.00	0.00	0.09	0.77
	**Sampling time**	**1,17**	**0.17**	**0.17**	**21.22**	**<0.001**
	**Sex × sampling time**	**1,17**	**0.04**	**0.04**	**4.64**	**<0.05**
Intracellular pH	Sex	1,17	0.00	0.00	1.93	0.18
	Sampling time	1,17	0.00	0.00	1.14	0.30
	**Sex × sampling time**	**1,17**	**0.02**	**0.02**	**13.98**	**<0.01**

Statistical significance was determined at *α* = 0.05 and is bolded.

**Table 7:** Summary of model selection results based on linear models predicting the cortisol, lactate, glucose, extracellular pH and intracellular pH concentrations of lake trout (*S. namaycush*) sampled at 0.5 h post-angling in the fall

Blood metric	Variable	Estimate	SE	*df*	*t*	*P*
Cortisol	**(Intercept)**	**−6763.64**	**962.80**	**4**	**−7.03**	**0.00**
	**Fish length**	**15.48**	**1.75**	**4**	**8.84**	**0.00**
	Fight time	10.27	8.68	4	1.18	0.30
	Air exposure	−9.81	2.71	4	−3.62	0.02
	**Angling depth**	**199.22**	**39.51**	**4**	**5.04**	**0.01**
	Barotrauma	−224.54	295.07	4	−0.76	0.49
Lactate	(Intercept)	34.69	12.85	4	2.7	0.05
	Fish length	−0.07	0.02	4	−2.95	0.04
	Fight time	0.27	0.12	4	2.30	0.08
	Air exposure	0.06	0.04	4	1.56	0.20
	Angling depth	−0.85	0.53	4	−1.62	0.18
	Barotrauma	0.22	3.94	4	0.06	0.96
Glucose	(Intercept)	10.02	3.29	4	3.05	0.04
	Fish length	−0.01	0.01	4	−0.84	0.45
	Fight time	0.03	0.03	4	0.93	0.41
	Air exposure	−0.02	0.01	4	−2.33	0.08
	Angling depth	0.27	0.13	4	2.02	0.11
	Barotrauma	−1.34	1.01	4	−1.33	0.25
Extracellular pH	**(Intercept)**	**7.22**	**0.36**	**4**	**20.28**	**0.00**
	Fish length	0.00	0.00	4	1.60	0.19
	Fight time	−0.01	0.00	4	−1.58	0.19
	Air exposure	−0.00	0.00	4	−1.23	0.29
	Angling depth	0.01	0.00	4	0.76	0.49
	Barotrauma	0.04	0.11	4	0.32	0.76
Intracellular pH	**(Intercept)**	**6.88**	**0.12**	**4**	**58.47**	**0.00**
	Fish length	0.00	0.00	4	2.35	0.08
	Fight time	0.00	0.00	4	2.07	0.11
	Air exposure	−0.00	0.00	4	−3.97	0.02
	Angling depth	0.00	0.00	4	0.32	0.77
	Barotrauma	−0.04	0.04	4	−1.08	0.34

Statistical significance was determined at Bonferroni-corrected *α* = 0.01 and is bolded.

Upon release, 6 fish displayed a “good” vigour score (4 females, 2 males) and 16 fish displayed an “excellent” vigour score (7 females, 9 males) after immediate sampling. For the 0.5-h sampling time, 8 fish displayed a “good” vigour score (5 females, 3 males) and 3 fish displayed an “excellent” vigour score (1 female, 2 males). Fish were observed to descend quickly to depth upon release with high ODBA levels before reaching a desired depth and remaining there with low ODBA levels. The time it took to reach maximum depth after release was not influenced by fight time or air exposure (Table 4). ODBA was significantly affected by sex and depth (Table 5), with males exhibiting higher activity overall (mean = 1.11 ± 0.01 opposed to female mean = 1.08 ± 0.01) and all fish exhibiting higher activity with increased depth (highest activity in <5 m).

## Discussion

We observed changes in reflexes, physiology and behaviour between seasons. In the summer, many fish displayed signs of reflex impairment and barotrauma. Barotrauma impairment was more pronounced in deeper water and larger fish. All 0.5-h blood indices were different than baseline values. Increased air exposure meant longer time to reach maximum depth. In the fall, we observed no reflex impairment. Symptoms captured in the barotrauma assessment (i.e. bloating of the abdomen, anal prolapse and haemorrhaging) are all likely a result of spawning (i.e. egg production) rather than pressure-related impairment, albeit it is possible that those signs could be exacerbated by capture from depth. Cortisol was 65.5% higher in females with all 0.5-h blood indices different than baseline values. Cortisol was also higher for larger fish and fish caught at deeper depths. Higher ODBA was observed for males and in shallower water, with the highest activity occurring in <5 m. These findings suggest that seasonal variations impact fish physiology and behaviour, with potential implications for their health.

### Mortality

In the summer, nine fish died during the 0-h sampling, and five deaths occurred within 0.5 h of holding (18.9% total mortality). No mortalities were observed during the fall sampling. No relationships were found between blood indices and mortality, but high surface water temperatures (mean summer temperature = 18.6°C, mean fall temperature = 13.15°C) and angling depth (mean summer depth = 26 m, mean fall depth = 3.37 m) were likely contributing factors. Water temperature and angling depth were higher in the summer; both are known stressors that can influence recovery following angling (Dinken *et al.,* 2022; Madden *et al.,* 2024; Robichaud *et al.,* 2024). For instance, Atlantic salmon show increases in mortality following angling when water temperatures exceed 18°C (Havn *et al.,* 2015), and other studies provide similar evidence in other salmonids (Meka and McCormick, 2005; Gale *et al.,* 2013; Sitar *et al.,* 2017). Aerobic performance of juvenile Ontario lake trout populations was reduced when acclimated above 19°C, and their critical thermal maximum was ~26°C when acclimated to 8°C (within range of approximate angling depth temperature in this study) (Kelly *et al.,* 2014). In our study, all mortalities occurred on days above 19°C, with the highest number of mortalities (eight fish) occurring when air temperatures reached 26°C. However, temperatures within preferred or optimal ranges may still increase angling mortality for many species (Gale *et al.,* 2013). Similarly, increased angling depth is more likely to induce barotrauma (see below) and has been shown to inhibit reflexes like orientation even after release (Madden *et al.,* 2024). Specifically for lake trout, mortality rates in our study were higher than those previously observed. Falk *et al.* (1974) observed a mortality rate of 6.98% while holding fish for up to 10 days in underwater holding cages in ~5 m of water (June to August). Loftus *et al.* (1988) observed a mortality rate of 14.9% while tethering fish to a buoy and line system that allowed them to move to their desired depth for ~48 h (summer season). Regardless, mortality rates for salmonids are commonly found to be less than 10% (Wydoski, 1977; Muoneke and Childress, 1994; Schisler and Bergersen, 1996). In our study, one fish died immediately upon capture and was the only fish to display oral organ eversion. For fish that died before they were released, it is possible that holding was a contributing factor. Fish were held at low densities (most often it was only one fish but occasionally we had a maximum of three) in a large, covered stock tank, and water was frequently exchanged. Surface water was transferred into the holding tanks, which may explain higher mortality rates because water temperature was higher than what fish would be exposed to if they were released and had been able to descend to their desired depth. Other studies have shown holding effects on fishes even when in low densities (Sopinka *et al.,* 2016; Mullen *et al.,* 2020; Howell *et al.,* 2023), although holding tanks are frequently used in field studies that assess post-capture mortality (Gale *et al.,* 2013). In addition to the angling itself, fish were exposed to a variety of other stressors associated with sampling that may have influenced mortality (i.e. blood sampling and handling). Our 0.5-h sampling point, in essence, represents a poor handling scenario in which fish are exposed to a culmination of stressors, some of which are inherent with studying impairment in many animals (Branson, 2008; Lawrence *et al.,* 2020).

### Reflex impairment

During the summer, lake trout experienced reflex impairment and barotrauma. Reflex impairment seems to be a common outcome of angling in lake trout and other salmonids (Kerr *et al.,* 2017; Brownscombe *et al.,* 2022). However, there is less evidence that lake trout experience pressure-related impairment following capture (Ng *et al.,* 2015; Gorman and Keyler, 2016; Sitar *et al.,* 2017; Howell *et al.,* 2023). Most studies exploring angling-induced barotrauma focus assessments on freshwater physoclists or deep-dwelling marine species (Morrissey *et al.,* 2005; McLennan *et al.,* 2014; Eberts *et al.,* 2018; Wegner *et al.,* 2021). Despite lake trout being physostomous, they displayed signs of barotrauma, which are in some cases serious signs. Reflex impairment and extent of barotrauma were significantly correlated, indicating that pressure-related impairment may also be a predictor of mortality. Loss of orientation and bloating of the abdomen were the most common impairments. They are similar in that they may impede fish from returning to depth and may prolong exposure to air and surface water temperatures as well as increase the likelihood of avian predation (Jarvis and Lowe, 2008; Raby *et al.,* 2014; Ferter *et al.,* 2015).

In the summer, fish were angled between 10 and 42 m, and barotrauma scores were significantly affected by total length, fight time and angling depth. Angling depth is well understood to impact barotrauma expression as water pressure is greater in deeper areas of a waterbody, resulting in more severe changes in external pressure upon capture (Butcher *et al.,* 2012; Hannah *et al.,* 2012; Kerwath *et al.,* 2013). Lake trout are known to exhibit phenotypic diversity with depth preferences (Zimmerman *et al.,* 2006; Stafford *et al.,* 2014). Larger fish may inhabit deeper sections of lakes and thus, when angled, are more susceptible to pressure changes (Sitar *et al.,* 2008). Additionally, characteristics of large fish responses to capture such as increased fight times may combine with pressure changes and influence impairment (Thorstad *et al.,* 2003; Reeves and Staples, 2011; Twardek *et al.,* 2018). Conversely, research on several fish species including smallmouth bass (*Micropterus dolomieu*), walleye (*Sander vitreus*) and yellow perch (*Perca flavescens*) in the St. Lawrence River found no effect of fish length or fight time on barotrauma impairment (Schreer *et al.,* 2009). However, these species inhabit shallower waters and are physoclistous, suggesting that their angling conditions and response to barotrauma may be different from that of physostomous species like lake trout.

Fish angled during the fall displayed no reflex impairment. There is limited knowledge on how angling impacts fish that are close to or have spawned. Reflex impairment was absent in 48% of sea-run brown trout (*Salmo trutta*) angled after spawning (Blyth and Bower, 2022). In another study, reflex impairment increased predictably with air exposure during angling treatments on pink salmon (*Oncorhynchus gorbuscha*) and chum salmon (*Oncorhynchus keta*) caught after their arrival to spawning locations (Raby *et al.,* 2013). In the present study, prolapse of the anus was the most observed manifestation of barotrauma across sampling times (0 h = 39.1% of fish, 0.5 h = 57.1% of fish). However, due to the comparatively shallow angling depths (mean = 3.37 m), the impairment almost entirely being displayed by females, and the fact that barotrauma impairment observed was mostly only anal prolapse, we believe our barotrauma assessment captured spawning-related injuries and anatomical changes rather than pressure-related impairment. It is possible that changes in pressure amplified the anal prolapse, but it is not possible to evaluate that supposition.

### Physiology

Angling induced physiological responses in lake trout regardless of season. The response we observed was typical for other studies on lake trout, salmonids and other fishes in general following angling (e.g. Milligan and Wood, 1986; Barton and Iwama, 1991; Donaldson *et al.,* 2014; Mullen *et al.,* 2020; McGarigal and Lowe, 2022). The absolute values in the blood indices also align with what others have found, such as cortisol and lactate in Arctic charr (*Salvelinus alpinus*) following a confinement stressor (e.g. Pottinger, 2010). However, we did not likely capture peak responses, so it is difficult to conclude the seriousness of the physiological response we observed. Fish must clear metabolites from the blood prior to regaining normal swimming performance (Milligan, 1996). The time that this requires can be context specific, with some studies showing recovery after 6 h or even 9 h post-capture (Arlinghaus *et al.,* 2009; Rapp *et al.,* 2012). Angling may result in metabolic and osmoregulatory disturbance that impair health and in extreme cases lead to mortality (Wedemeyer and Wydoski, 2008; Holder *et al.,* 2022). That said, none of the blood indices in our study were predictors of mortality, so at least after 0.5 h, the physiological response in lake trout captured in the summer do not seem to be detrimental to fish survival.

In the fall spawning period, at the 0.5-h sampling point, cortisol was 65.5% higher in females, suggesting that their baseline levels differ from males. In Pacific salmon, females are distinct from males in that they are less capable of responding to environmental stressors (Sandblom *et al.,* 2009; Clark *et al.,* 2011) and generally display higher levels of cortisol (Pottinger *et al.,* 1996; Donaldson *et al.,* 2010). The sex hormone 17β-estradiol is important for fish reproduction (Piferrer and Donaldson, 1994; Jeffries *et al.,* 2012; Sinchak and Wagner, 2012) and may become reduced in response to angling stressors (Donaldson *et al.,* 2014). Reduction in 17β-estradiol may in turn influence reproductive output through less energy allocated into egg production. Due to high maternal investment in gonadal development (Jastrebski and Morbey, 2009; Johnston, 2018), and differing baseline values of certain blood parameters, lake trout females are likely more susceptible to angling-related stressors than males (via increased allostatic load). Allostatic load (i.e. the cumulative effects of stress) is greater for female fish, with females also displaying elevated levels of lactate and glucose (Jeffries *et al.,* 2012). Increased lactate levels are generally associated with fight time (Meka and McCormick, 2005; Twardek *et al.,* 2018; Blyth and Bower, 2022), which is to be expected due to lactate being a by-product anaerobic exercise (Milligan and Wood, 1986).

Lactate was not significantly higher in females than in males. Female salmonids have been shown to have lower aerobic scope than males (Clark *et al.,* 2011) and therefore may have higher circulating lactate levels to account for increased usage of anaerobic metabolism. Lactate dehydrogenase can fuel anaerobic metabolism, and female sockeye salmon rely more heavily on anaerobic metabolism during exhaustive exercise (Burnett *et al.,* 2014). These fish also display higher levels of cardiac lactate following handling (Eliason *et al.,* 2020). While lake trout do not display the same degree of morphological difference between sexes, the process of spawning (i.e. egg production) may inherently impact the allocation of metabolites (Lahnsteiner *et al.,* 1999). Further research should investigate the physiological differences between male and female lake trout during reproduction, since lake trout do not display the same migration or reproductive behaviours as the more studied Pacific salmons (Esteve *et al.,* 2008; Farrell *et al.,* 2008; Binder *et al.,* 2015; Raby *et al.,* 2016). Additionally, growth, locomotion and reproduction may diminish as aerobic scope decreases at lower temperatures (Clark *et al.,* 2013). Conceptually, lake trout are forced to use anaerobic metabolism during fall angling and thus may take longer and use more resources to recover than during a period where their aerobic scope is not contracted. Therefore, it is surprising that elevated lactate was not observed in females in this study.

### Locomotor activity

In both seasons, fish descended quickly upon release and remained at a similar depth for the remaining monitoring period unless barotrauma signs were present. Quick descent followed by a period of reduced activity has been seen in other species such as Pacific cod (Nichol and Chilton, 2006). In our study, the time to reach maximum depth was impacted by fight time and air exposure in the summer but not in the fall. Extended activity during the beginning of an angling event may leave little energy reserved for post-release escape. While not statistically significant, fish with barotrauma (which was driven by angling depth) appeared to exhibit higher ODBA levels on the surface of the water, likely indicating their continued activity as a result of attempting to return to depth. Maintaining normal swimming behaviours is critical to fish recovery, and impairments that inhibit the return to depth or force fish to float at the surface can accelerate mortality (Gravel and Cooke, 2008; Drumhiller *et al.,* 2014). Swimming activity was influenced by angling depth in the summer and both angling depth and sex in the fall. Higher ODBA was likely associated with shallow depths due to fish displaying a burst of energy to escape once returned to the water.

### Management implications and conclusions

Our study builds upon knowledge surrounding the susceptibility of freshwater fish to recreational angling in deep water, specifically showing susceptibility of a physostomous species and differences between sexes during fall spawning. Clearly, water temperature is a concern when angling during the summer, and thus, best practices to limit impacts of angling on fish during this time should be prioritized. Larger “trophy” sized fish displayed higher cortisol in the fall and more barotrauma in the summer. Likewise, angling depth increased the barotrauma score in the summer, suggesting that anglers should avoid targeting lake trout in deep water and/or consider using descending devices. In addition to this and especially for larger fish, anglers should employ best practices such as limiting air exposure, fight times and handling so fish have sufficient energy to be able to attain depths needed to abate some of the barotrauma symptoms.

The Clearwater Lake population of lake trout provides eggs and milt for stocking lakes across Manitoba and other parts of Canada (K. Dyck, personal communication, April 19, 2023). Ensuring population numbers remain high and that fish do not experience negative effects associated with the recreational fishery is important. During the spawn, when fish inhabit shallow depths, we did not observe mortality or reflex impairment, and signs of barotrauma were minimal. However, fish showed signs of sub-lethal effects and female responses were different than males. The sensitivity of adult fish during spawning and the effects of broodstock collection methods on embryo viability continue to be of interest for fisheries management (Smukall *et al.,* 2019; Duncan *et al.,* 2023). Aerobic scope is not well understood during reproduction, thus studying how aerobic scope may be impacted by the process of spawning would provide insight into potential effects on fish recovery following angling. In addition to this, effects of angling stress on individual gametes, subsequent zygote development and long-term resilience of offspring would allow for quantification of broad sub-lethal effects on population growth. Angling may coincide with the spawning period of multiple species, and thus, understanding the impacts of such stressors is critical to protecting populations (Bade *et al.,* 2019; Tufts *et al.,* 2019).

## Supplementary Material

Supplementary_material_coae041

## Data Availability

Data will be made openly available upon request.
