# Supplementary material for: Sex and season influence behaviour and physiology of lake trout following angling
Source: Conserv Physiol. 2024 Jul 5;12(1):coae041. doi: 10.1093/conphys/coae041 (PMC11224997; doi:10.1093/conphys/coae041)
Supplement: Supplementary_material_coae041 [file supplementary_material_coae041.pdf]

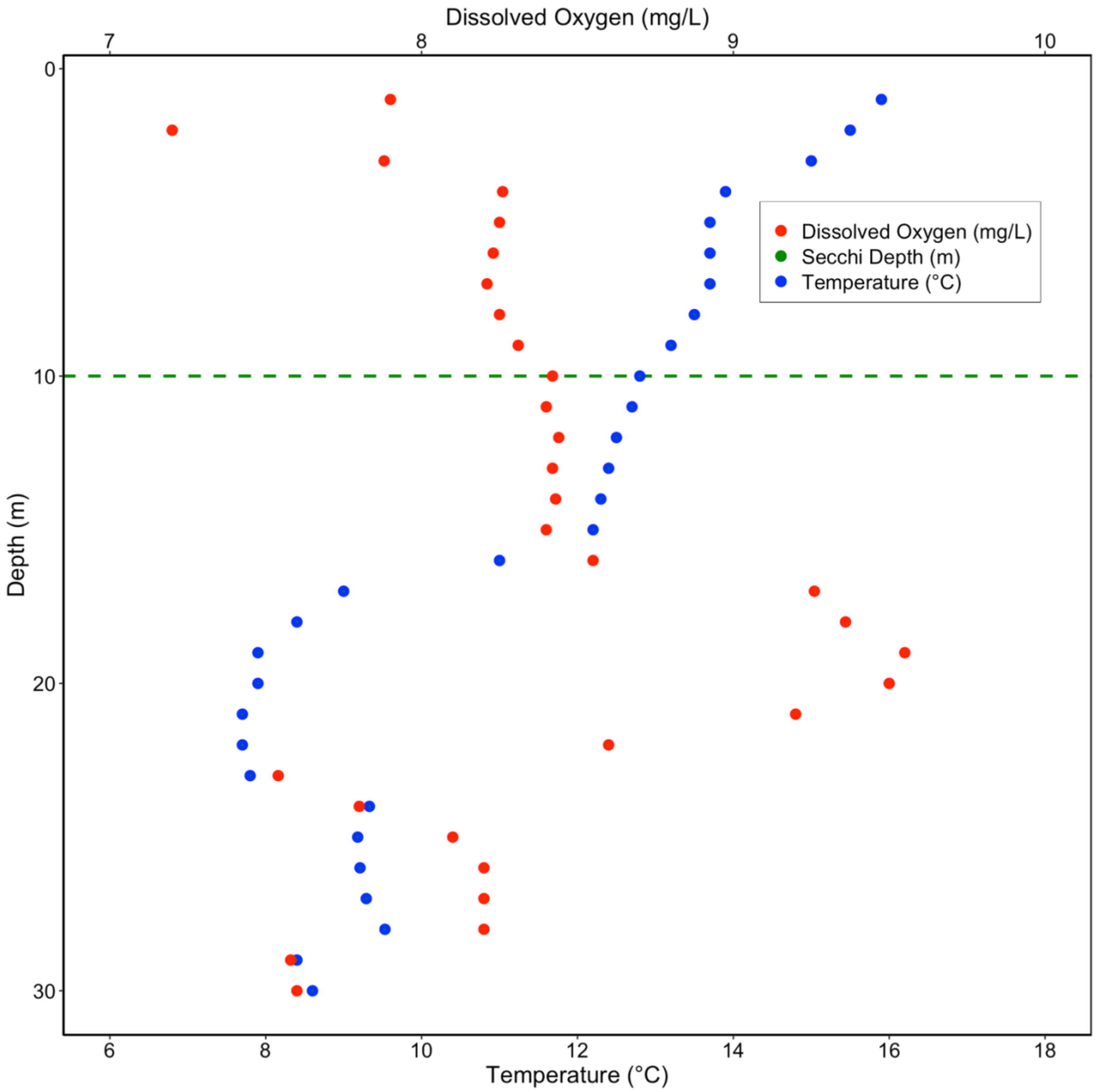


Figure S1. Vertical profile of Clearwater Lake (54.0570° N, 101.0564° W) showing water temperature (°C) as blue dots, dissolved oxygen concentration (mg/L) as red dots, and secchi depth as a green dashed line. Maximum depth of the lake is 39 m. Data was collected on July 5, 2022.

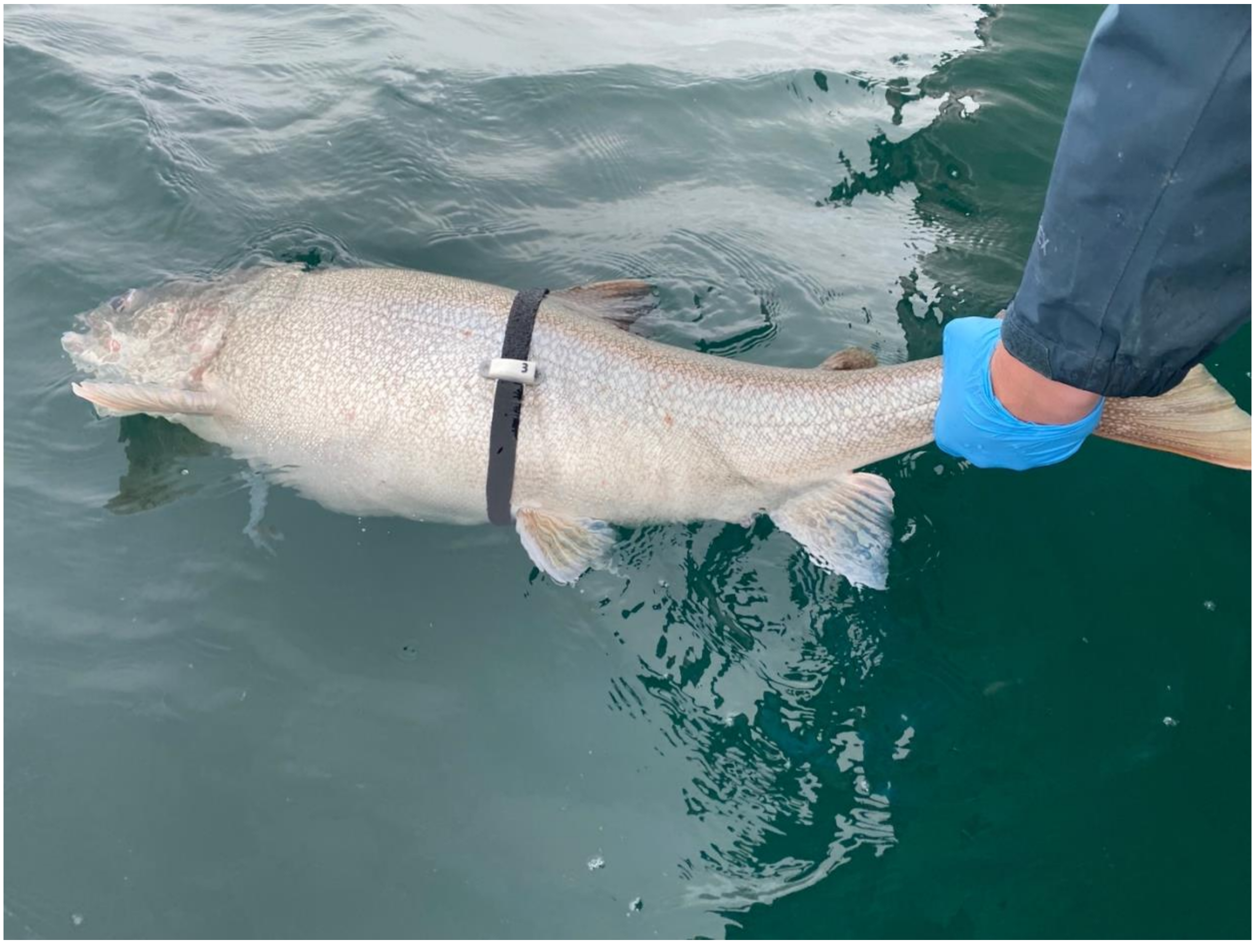

Figure S2. Placement of a tri-axial accelerometer harness on a lake trout (*Salvelinus namaycush*) to quantify behaviour following angling. The accelerometer was bonded to a waterproof 3D-printed plate using marine epoxy and then threaded onto a section of Velcro tape that was fastened anterior to the dorsal fin with the accelerometer on the lateral side above the left pectoral fin.
